# Enhancement of human sodium iodide symporter gene therapy for breast cancer by HDAC inhibitor mediated transcriptional modulation

**DOI:** 10.1038/srep19341

**Published:** 2016-01-18

**Authors:** Madhura G. Kelkar, Kalimuthu Senthilkumar, Smita Jadhav, Sudeep Gupta, Beyong-Cheol Ahn, Abhijit De

**Affiliations:** 1 Molecular Functional Imaging Lab, ACTREC, Tata Memorial Centre, Kharghar, Navi Mumbai, India; 2 Department of Nuclear Medicine, Kyungpook National University, Republic of Korea; 3 Department of Medical Oncology, Tata Memorial Hospital, Parel, Mumbai, India

## Abstract

The aberrant expression of human sodium iodide symporter (NIS) in breast cancer (BC) has raised the possibility of using targeted radioiodide therapy. Here we investigate modulation of endogenous, functional NIS expression by histone deacetylase inhibitors (HDACi) *in vitro* and *in vivo*. Luciferase reporter based initial screening of six different HDACi shows 2–10 fold enhancement of NIS promoter activity in majority of the cell types tested. As a result of drug treatment, endogenous NIS transcript and protein shows profound induction in BC cells. To get an insight on the mechanism of such transcriptional activation, role of Stat4, CREB and other transcription factors are revealed by transcription factor profiling array. Further, NIS-mediated intracellular iodide uptake also enhances substantially (p < 0.05) signifying functional relevance of the transcriptional modulation strategy. Gamma camera imaging confirms 30% higher uptake in VPA or NaB treated BC tumor xenograft. Corroborating with such functional impact of NIS, significant reduction in cell survival (p < 0.005) is observed in VPA, NaB or CI994 drug and ^131^I combination treatment *in vivo* indicating effective radioablation. Thus, for the first time this study reveals the mechanistic basis and demonstrates functional relevance of HDACi pre-treatment strategy in elevating NIS gene therapy approach for BC management in clinic.

The current focus for development of targeted strategies in breast cancer (BC) is actively being researched to identify suitable therapy procedures that can eliminate BC cells with high specificity while minimizing the side effects. In this context, the aberrant over-expression of human sodium iodide symporter (NIS) protein in breast cancer tissue is gaining great deal of attention. Being a member of the solute carrier transporter (SLC5A5), NIS is an intrinsic plasma membrane glycoprotein that mediates active iodide transport in thyroid follicular cells. NIS mediated iodide transport is also seen in extra-thyroidal tissues such as salivary gland, gastric mucosa and lactating mammary tissue where NIS is differentially regulated or subjected to distinct post-translational modifications that are not entirely understood^1^,^2^. As an endogenous protein, NIS function can be visualized using gamma or positron emitting isotopes such as ^99m^Tc, ^125^I or ^124^I respectively. The same protein can also be applied for therapy purposes using beta- or alpha-emitting isotopes like ^131^I, ^186^Re, ^188^Re and ^211^At^3^,^4^. Thus endogenous NIS-mediated radioiodide therapy is a gene-targeted, inexpensive method with relatively lesser side effects as can be revealed by years of practice in thyroid cancer clinic.

The pioneering study by Tazebay *et al*. showed aberrant over-expression of NIS in breast malignancies over normal breast tissue indicating its potential role for BC management^5^. Further study using ^99m^TcO4 has confirmed functional NIS expression in human malignant mammary tissues^6^. NIS-mediated iodide uptake in BC metastatic nodules has also been confirmed by scintigraphic procedure while suppressing thyroidal iodide uptake using T3 and methimazole^7^. Culminating data from all these literatures indicate that NIS-mediated radioiodide imaging and therapy methods could potentially be used as an alternative approach or in combination with other existing methodologies towards management of the malignant breast disease. Recently our group has also reported analysis on BC subtype specific intensity profiling of NIS expression in a relatively large dataset^8^. The results revealed that while ~70% of the BC cases are positive for NIS expression, about 30% of NIS positive (2+ and 3+ score) cases showed intense staining equivalent to thyroid or salivary gland expression. Therefore, to use this gene-targeted radioiodide therapy in patients exhibiting moderate or low scores (i.e. lesser than 2+ score), defining ways to elevate this endogenous target expression can impact NIS based therapy procedure.

Various groups around the world have initiated research looking for ways to modulate endogenous target expression of NIS in thyroid, mammary or other tissue targets. NIS gene expression is differentially regulated in thyroid and breast tissue and potential regulators of NIS expression in breast tissue are not well characterized. Several groups have reported that NIS in BC cells can be induced *in vitro* using lactogenic hormones, insulin and even by some nuclear receptor ligands, such as retinoids and peroxisome proliferator-activated receptor-γ (PPARγ) ligands^2^,^9^,^10^,^11^. All-trans retinoic acid (atRA) alone or in combination with other glucocorticoids has been demonstrated to induce both NIS gene expression as well as iodide accumulation in MCF-7 cells and *in vivo* mouse model^12^,^13^. Even though these findings suggest their potential clinical use, to date preclinical or clinical efficacy is not yet proven.

Histone deacetylase inhibitors (HDACi) are known for exerting epigenetic control by regulating chromatin structure and gene expression. Additionally, HDACi can also modulate variety of cell functions such as growth, differentiation and survival by affecting non-histone proteins such as transcription factors, molecular chaperones and structural components^14^,^15^. Similarly, it is also repoted that NIS expression can be modulated by certain HDACi in thyroid cells even though their exact molecular mechanisms are not understood^16^,^17^. Very recently, reports have shown the effect of HDACi on BC cells as well^18^,^19^. Since NIS gene regulation in thyroid and breast tissue is differentially regulated, studying HDACi mediated modulation of NIS expression and function *in vivo* are of great interest. Thus, in the present study, we have performed a comprehensive investigation to reveal biochemical basis of HDACi mediated modulation of NIS expression and function in BC cell and animal model. The study implicates that epigenetic transcriptional modulation strategy as a promishing approach, which may be extended for clinical trial in near future.

## Results

### Pan-HDAC inhibitors representing various chemical classes enhance NIS promoter activity in breast cancer cells

**Figure 1:**
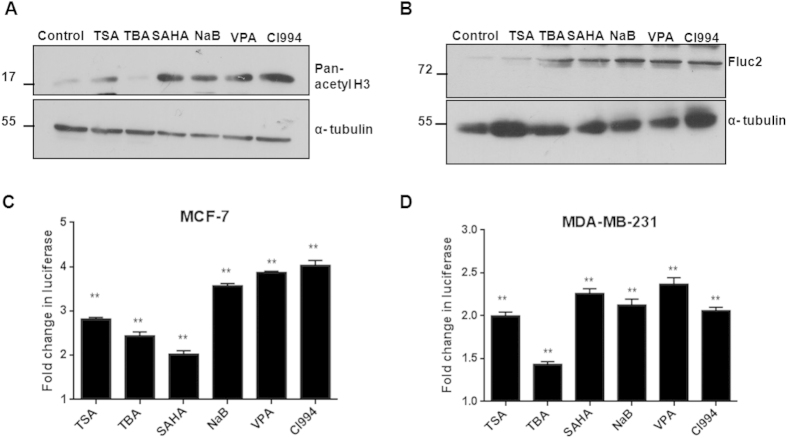
HDACi drugs significantly enhance human NIS promoter activity. (**A)** The target effect of HDACi on histone H3 acetylation in MCF-7 cells. Cells exposed to HDACi for 48 hours were assessed by western blotting using pan-acetyl-H3 monoclonal antibody and α-tubulin as loading control. (**B)** Western blot analysis of luciferase reporter induction using Fluc antibody in engineered MCF-7 pNIS-Fluc2.TurboFP cells. Full length western blots for the acetyl-H3 and Fluc2 were shown in supplementary Fig. 8A,B respectively. (**C,D)** Charts representing reporter signal fold change over untreated control (**indicates high significance p < 0.005, ns indicates non-significant) as a measure of NIS promoter activity upon HDACi treatment. Both MCF-7 and MDA-MB-231 engineered cells stably over-expressing pNIS-Fluc2.TurboFP reporter construct were exposed to various HDACi for 48 hours and luciferase activity was measured.

Six different HDACi i.e. Trichostatin A (TSA), Sodium butyrate (NaB), Valproic acid (VPA), Suberoylanilide hydroxamic acid (SAHA) and Tubastatin A (TBA) representing various chemical classes (Table 1) were tested for NIS promoter transcription modulation in multiple BC cell lines. We have included receptor positive MCF-7 as well as receptor negative MDA-MB-231 cells over-expressing NIS promoter-reporter (pNIS-Fluc2.TurboFP) plasmid. The target effect of HDACi drugs was tested in MCF-7 cell line revealing increased histone H3 acetylation except for TBA, which is a known HDAC6 specific inhibitor^20^ (Fig. 1A). Further the minimal drug dose requirement to promote NIS gene expression was determined by luciferase reporter assays against increasing concentration of each drug using the established MCF-7 cell line expressing pNIS-Fluc2.TurboFP (Supplementary Fig. 1). Cytotoxicity assessment was also done using a concentration dependent cell survival analysis of both MCF-7 and MDA-MB-231 cell lines (Supplementary Fig. 2). The same minimal drug concentration (~IC_70_ equivalent) was further used for all successive promoter regulation experiments. Candidate drug effects on engineered MCF-7 cells showed significantly higher Fluc2 expression as reveled by western blot analysis (Fig. 1B). Further, luciferase reporter activity also confirmed a 2–4 fold enhanced photon output in MCF-7 and a 1.4–2.4 fold increment in MDA-MB-231 cells (Fig. 1C,D). The fold gain in reporter activity upon drug treatment was found to be significantly higher (p ≤ 0.005) when compared to the untreated control in these cells. The same reporter system was also tested by transient transfection method in two additional cell lines i.e. Zr-75-1 (hormone receptor positive) and MDA-MB-468 (hormone receptor negative) and similar trend of higher fold induction of normalized luciferase activity have been observed (Supplement Fig. 3A). The point to note here is that, of the various drugs tested, NaB, VPA and CI994 showed consistent gain in reporter activity across the cell lines. Inhibition with TBA, a HDAC6 specific inhibitor, showed around 1.5–2 fold gain in reporter activity in hormone receptor negative cell lines, while TSA and SAHA showed heterogeneous fold gain in reporter activity across all the cell types tested. Additionally, the NIS promoter specific effect of HDACi treatment was proven by testing other ubiquitous promoters like CMV or chicken beta-actin promoter (CAG), where no radical change in reporter activity noted (Supplementary Fig. 4). Considering HDACi mediated up-regulation of NIS gene can have broader scope in thyroid and other cancer types, the possibility was tested by using several additional human cancer cell lines such as NPA and ARO (thyroid cancer), A2780 (ovarian cancer) and HT1080 (fibrosarcoma) over-expressing the pNIS-Fluc2.TurboFP plasmid. Candidate HDACi drug effects of SAHA, VPA and CI994 at 48 hours revealed that except for the CI994 in thyroid cancer cell lines, all other non-breast cancer cell lines showed significant increase in NIS promoter activity (Supplementary Fig. 3B). Together, these results suggest that the effect of HDAC inhibition may lead to NIS promoter activation by known mechanism of increased DNA binding ability, but for certain HDACi candidate the NIS promoter activation in breast cancer cells may happen through independent (or tissue-specific) activation of factors than their thyroid counterparts.

### HDAC inhibition enhances endogenous NIS gene expression through Stat4, CREB and other transcription factors (TF) in breast cancer cells

**Figure 2:**
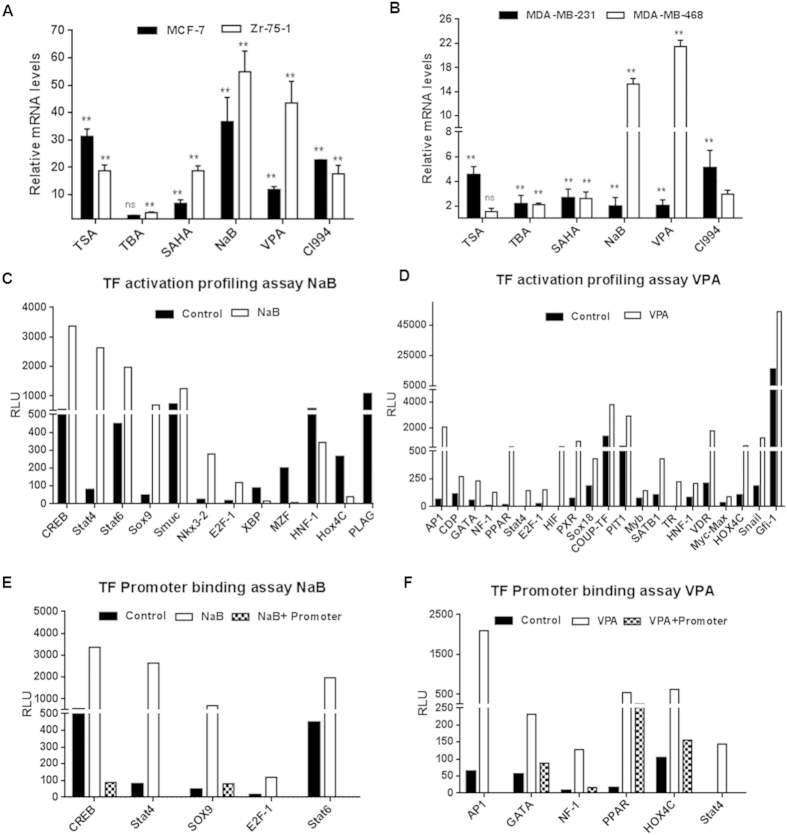
Induction of endogenous NIS transcript after HDACi treatment in BC cell lines. (**A,B)** MCF-7, MDA-MB-231, Zr-75-1 and MDA-MB-468 cells treated with different HDACi for 48 hours were evaluated for NIS mRNA by real-time PCR using ABI probe. Results were normalized for housekeeping gene GAPDH and presented as relative fold differences to untreated control (**indicates high significance p < 0.005, ns indicates non-significant). (**C,D)** Graphs showing activation of transcription factors (TFs) post NaB (**C**) or VPA (**D**) treatment in MCF-7 cell line. TFIID was used for normalization as per manufacturer’s guideline. Candidate TFs showing greater or lesser than 1.5 folds difference in luminescence read-outs after normalization (threshold cut-off) were plotted. (**E)** Graph represents TF binders to human NIS promoter upon NaB treatment identified by promoter binding array. In comparison to drug treated nuclear extract samples, when NIS promoter is added, the luminescence read-out is lowered due to competitive binding to the promoter. Candidates like CREB, Stat4, Stat6, Sox9 and E2F-1 showed significant binding efficiency to the promoter. (**F**) TF binders identified such as AP1, GATA, NF-1, PPAR, Hox4C and Stat4 upon VPA treatment using the same array.

Based on the results obtained from the reporter based system, we were encouraged to see if the same treatments corroborate in endogenous NIS activation. Therefore, we isolated cDNA from drug treated and untreated MCF-7, Zr-75-1, MDA-MB-231 and MDA-MB-468 cells and assessed NIS transcript using Taqman real-time PCR probe for human NIS and GAPDH as housekeeping control. As shown in Fig. 2A,B, each data point represents normalized transcript value relative to their untreated control. Overall, variable increment of NIS transcript (2–55 folds) was observed across all cell types tested (p ≤ 0.005). In particular, NaB and VPA treatment showed highest fold change of NIS mRNA expression, while TBA treatment proved to be least effective. Point to be indicated here is that hormone receptor positive MCF-7 and Zr-75-1 cell lines showed much higher transcript levels (~3–10 fold) than the receptor negative cells (MDA-MB-231 and MDA-MB-468). Further, fold induction in NIS transcript expression under drug influence was also observed in the non-breast cancer cell lines NPA and ARO, HT1080 and A2780 (data not shown).

To gain further insight into the mechanisms leading to NIS up-regulation, bioinformatics analysis of the NIS promoter sequence using the Transfac and Genomatix softwares revealed the availability of ~64 putative transcription factor (TF) binding sites. Following this analysis, we performed TF activation array for 96 global TF signatures to identify differentially regulated TFs under NaB and VPA drug influence in MCF-7 cells and compared to the untreated cells. Of these 96 common TF candidates present in the array, ~56 candidates were found in common where at least one putative DNA binding site available on human NIS promoter. As revealed from the array data, HDAC inhibition by NaB markedly increased (>1.5 fold) expression of 7 TFs i.e. CREB, Stat4, Stat6, Sox9, Smuc, Nkx3.2 and E2F-1, whereas 5 others like XBP, MZF, HNF-1, Hox4C, and PLAG exhibited suppression (< −1.5 fold) (Fig. 2C). The same cell line when checked for VPA induced TF activation, 21 factors showed higher than 1.5 fold activation (Fig. 2D), but no significant suppression of any factor. Only Stat4 and E2F-1 were found to be activated in common with those under NaB influence.

Further, the ability of TF binding to the NIS promoter was verified using promoter binding array where NIS promoter DNA was added in the plate together with the nuclear extract of MCF-7 cells treated or untreated with NaB (Fig. 2E) or VPA (Fig. 2F). Promoter binding array results confirmed that 5 of the 7 TFs bind under NaB treatment and for VPA treatment 6 out of 21 factors bind significantly to the NIS promoter. Stat4 was the only factor found in common that binds to the NIS promoter. Together these findings demonstrate possible role of TFs like Stat4, CREB, Nkx3.2, AP1 etc. majorly influence NIS expression in HDACi treated MCF-7 cells (summarized in Table 2).

### HDAC inhibitor treatment significantly impacts NIS function in breast cancer cells

**Figure 3:**
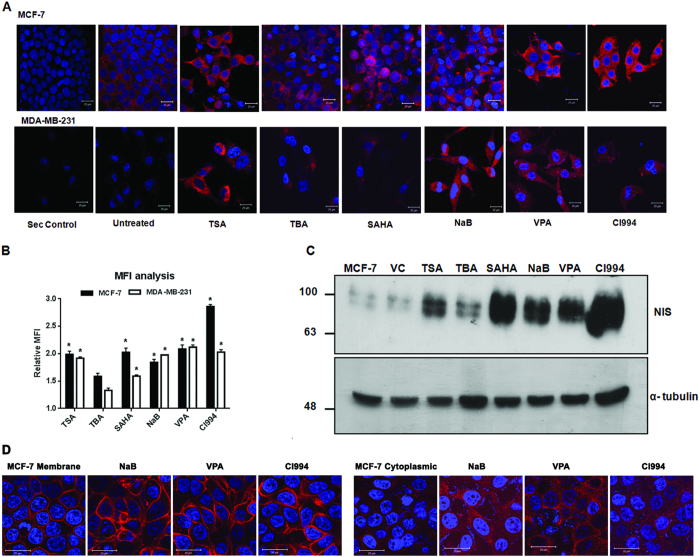
Enhancement of NIS protein by HDACi treatment in breast cancer cells. **(A)** Immunofluorescence photographs of MCF-7 and MDA-MB-231 cells showing marked increase in NIS protein content when treated with various HDACi. Drug treated and untreated cells were probed with NIS monoclonal antibody and detected with Dylight 633 secondary antibody (red). A secondary antibody control was also included in the study. Cell nuclei were cross stained using DAPI (blue). Scale bar represents 20 μm. (**B)** Graph showing fold increase in mean fluorescence intensity (MFI) of NIS staining after HDAC inhibition in MCF-7 and MDA-MB-231 cells. Average MFI was calculated from 30–50 cells using LSM Image browser and fold difference with respect to untreated cells plotted. (**C)** Western blot showing NIS protein content in MCF-7 cells after different HDACi treatments. DMSO was used as a vehicle control (VC). Total 60 μg of protein was loaded in each lane and alpha-tubulin was used as an endogenous control. Full length western blot for NIS protein was shown in supplementary Fig. 8C. (**D**) NIS immunofluorescence photographs of MCF-7 clonal cells differentially over-expressing NIS protein either at the membrane (left panel) or at the cytoplasm (right panel) treated with NaB, VPA and CI994 drugs.

Taking the study more towards functional validation, first we have verified if enhanced endogenous NIS transcript under HDACi drug influence successfully translates into enhanced protein expression. A 2–3 folds increase in NIS protein content was recorded in both MCF-7 and MDA-MB-231 cells after majority of the HDACi treatment except for TBA (Fig. 3A,B). Enhanced protein production was further evidenced by performing western blotting using human NIS monoclonal antibody (Fig. 3C). Using MCF-7 clonal cell lines, differentially over-expressing NIS protein either at the membrane or at the cytoplasm^21^, we have also checked if the effect of NaB, VPA and CI994 drugs alter the NIS protein localization in cells (Fig. 3D). Together, these results suggest that except for HDAC6 specific inhibitor (TBA), other pan-HDAC inhibitors tested here can significantly induce endogenous NIS protein expression without influencing the protein localization in the cells.

**Figure 4:**
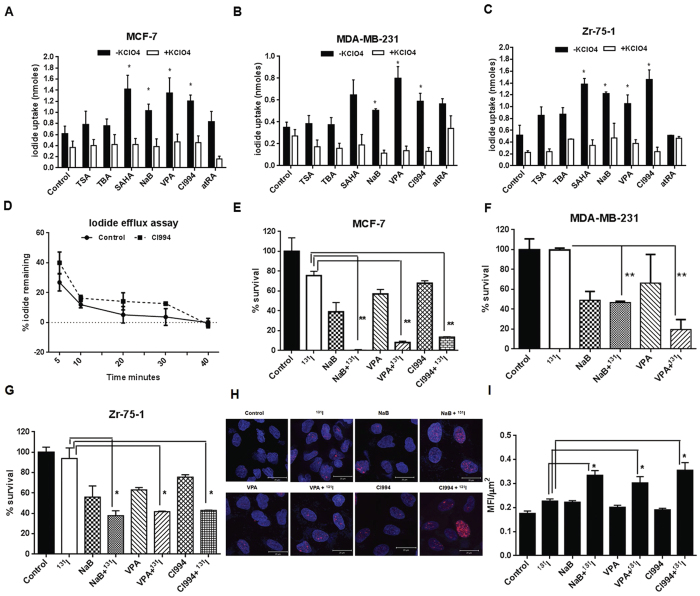
Modulation of NIS function by HDACi in BC cells. (**A**–**C)** Effect of the HDACi drugs and atRA on NIS-mediated iodide accumulation in MCF-7, MDA-MB-231 and Zr-75-1 cells respectively. 30 μM potassium perchlorate was used for blocking iodide uptake. The Y axis scale bars represent nanomoles (nmoles) of iodide uptake after 48 hours of HDACi and atRA treatment of cells. Error bars indicate standard error of mean. (**D**) Chart showing iodide efflux assessment done using MCF-7 cells. CI994 drug was used as a candidate HDACi representative to verify change in efflux upon drug treatment. (**E**–**G)** Charts represent measure of percentage cell survival after selective killing by ^131^I treatment in the presence or absence of candidate HDACi treatment as marked. MCF-7, MDA-MB-231 were exposed to 50 μCi/ml of ^131^I and Zr-75-1 cells were exposed to 100 μCi/ml of ^131^I with or without pre-treatment of NaB/ VPA/ CI994. Cell survival was measured by their colony forming ability (**indicates high significance p < 0.005). (**H**) Immunofluorescence photographs showing DNA damage response after ^131^I exposure detected by γ-H2Ax foci formation. Zr-75-1 cells were exposed to 100 μCi/ml ^131^I with or without pre-treatment of NaB/ VPA/ CI994 and probed with γ-H2Ax antibody after fixation. Foci were detected by Dylight 633 secondary antibody (red). Cell nuclei were cross stained using DAPI (blue). Scale bar represents 20 μm. (**I)** Graph showing MFI/μm^2^ of foci formation after ^131^I exposure with or without HDACi pre-treatment. Average MFI of 35 cells was calculated using LSM image browser and plotted.

As evidenced from many clinical literatures, high NIS mRNA or protein expression may not necessarily result in higher iodide uptake^22^. Therefore, we then verified the NIS functional modulation by HDACi treatment in cultured cells. BC cells treated with the same set of six HDACi were tested for their functional significance by iodide uptake assay. The all trans retinoic acid (atRA) was used as a known inducer of NIS function in breast cancer cell lines. In line of previous reports, we have recorded significant higher iodide uptake in MCF-7 and Zr-75-1 cells, but only a marginal increase in MDA-MB-231 cells after 24 hours of treatment with 1 μM atRA (Suppl. Fig. 5). Interestingly, the uptake in response to atRA reduces significantly at 48 hours. On the other hand, the perchlorate sensitive NIS-mediated iodide uptake increased up to 1.7–2.8 folds in MCF-7 and Zr-75-1 cell lines at 48 hours and it remain nearly unchanged at 24 hours of HDACi drug treatment. The iodide uptake in MDA-MB-231 cells was found to be much lower (Fig. 4A,C). When compared to the untreated cells, NaB, VPA, SAHA and CI994 treatment showed consistent and significant (p < 0.05) increase in iodide uptake in both MCF-7 and Zr-75-1 cells, and around 1.5–2 fold induction in uptake was noted with NaB, VPA and CI994 drug treatments in MDA-MB-231 cell line. TSA and TBA treatment resulted in marginal increase across all cell types tested. Further, as the steady-state accumulation is the net outcome of iodide uptake and iodide efflux, iodide retention time being another important parameter for achieving good therapeutic efficacy. Therefore, we performed iodide efflux assay using untreated and CI994 treated MCF-7 cells. Compared to the untreated cells, where 88% of accumulated iodide was released during first 10 minutes, CI994 treated cells showed similar 84% release in 10 minutes (Fig. 4D).

### NaB/VPA/CI994 treatment augments ^131^I mediated radioablation in breast cancer cells

Therapeutic efficacy of ^131^I radioablation was verified in MCF-7, MDA-MB-231 and Zr-75-1 cell lines with variable endogenous NIS protein content and survival assessment was done by clonogenic assay (Fig. 4E–G). Since the judgment to make here is whether HDACi treatment affects survival fraction, first the minimal amount of ^131^I required was determined. In case of MCF-7 and MDA-MB-231 50 μCi/ml and for Zr-75-1 100 μCi/ml was found to be sufficient for more than 75% cell survival. In comparison, drug treated cells when exposed to the same amount of ^131^I, highly significant reduction in the cell survival (p ≤ 0.005) was observed in all three cell lines. In the presence of perchlorate, HDACi and ^131^I treated MCF-7 cells survived to a greater extent indicating NIS specific radioablation (Suppl. Fig. 6). The reduced survival percentage of cells against minimal ^131^I radioactivity used further assures that enhanced iodide symporter function by HDACi pre-treatment can be proved to be useful for this gene therapy approach. We have also confirmed specific radiation injury caused in Zr-75-1 cells by staining for γ-H2Ax foci that forms due to DNA double strand break (Fig. 4H,I). Significant enhancement (p ≤ 0.01) in the number of γ-H2Ax foci was observed in cells when they are treated with all three HDAC inhibitors than cells treated with radiation alone.

### Low dose HDACi pre-treatment *in vivo* improves radiotracer uptake and ^131^I therapy efficacy in breast cancer tumor xenograft

**Figure 5:**
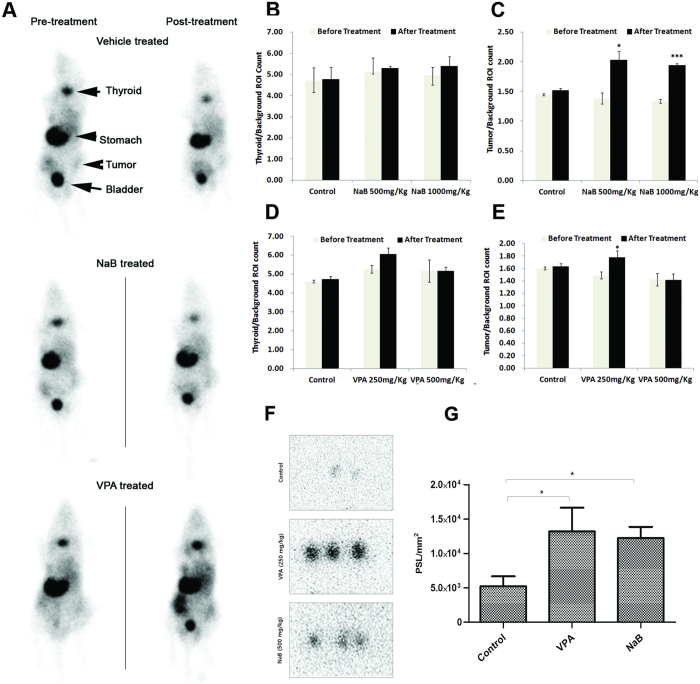
Preclinical validation of NIS functional augmentation by NaB and VPA treatment. (**A**) Accumulation of ^99m^Tc-pertechnetate in NIS-expressing tissues like thyroid, gastric mucosa (stomach) and MDA-MB-231 tumor xenografts pre- and post-NaB and VPA treatment was recorded by gamma camera imaging 20 minutes after radiotracer injection. Mice untreated or treated with lower dose of the candidate drugs i.e. 250 mg/kg for VPA and 500 mg/kg of NaB tested are represented. (**B,C**) Charts representing normalized ^99m^Tc-pertechnetate uptake (count) measured from thyroid (**B**) and tumor (**C**) tissue of mice images untreated or treated with two different concentration of NaB. (**D,E**) Chart representing similar uptake values upon VPA treatment. **F**. Digital autoradiography images showing intra-tumor uptake of ^125^I at 4 hours after tracer injection. Images represent MDA-MB-231 tumor tissue sections which were grown on nude mice treated as vehicle control, VPA (250 mg/kg) and NaB (500 mg/kg) as marked. (**G**) ROI analysis from autoradiograph images above. The quantitative results are expressed as PSL/mm2 (mean ± SD) and *indicate p < 0.05.

In order to validate our *in vitro* and cell culture findings, we performed noninvasive gamma camera imaging to first study the effect of NaB or VPA on NIS-mediated ^99m^Tc-pertechnetate uptake in MDA-MB-231 xenograft model. The results reveal 30% higher accumulation of radiotracer in tumor in VPA treated cases than in vehicle control. These results indicate that a minimally toxic lower dose of NaB or VPA can effectively enhance NIS expression *in vivo* resulting in increased uptake value. Besides tumor uptake, high radiotracer uptake was also seen in tissues such as stomach and thyroid where NIS protein is naturally present for physiological reasons (Fig. 5A). Interestingly, treatment with NaB (500 mg/kg and 1000 mg/kg tested) shows higher ^99m^Tc-pertechnetate accumulation in tumor but not in the thyroid of the mice (Fig. 5B,C). Similarly, by computing normalized tumor and thyroid uptake in mice, significant increase in tumor uptake was observed after 250 mg/kg VPA treatment but not 500 mg/kg dose (Fig. 5D,E). Since these drugs at the delivered dose are expected to show some anti-proliferative effect on the tumor cells, expected reduction of tumor size may have complicated the net radiotracer uptake at the tumor site. Autoradiography of NIS mediated radio-iodine uptake in MDA-MB-231 tumor xenograft clearly displayed much higher accumulation of ^125^I in treated group of mice (Fig. 5F,G). The ROI analysis of autoradiograph image has confirmed significantly higher uptake in VPA (250 mg/kg) and NaB (500 mg/kg) treated mice than in untreated control group. To further support *in vivo* uptake studies, we checked NIS protein content in untreated and VPA treated Zr-75-1 tumor xenograft tissue by immunohistochemistry (IHC). As shown in Supplementary Fig. 7, digital IHC analysis of these samples revealed significant enhancement of NIS-immunopositivity (p = 0.014) in 250 mg/kg VPA treated tumor.

**Figure 6:**
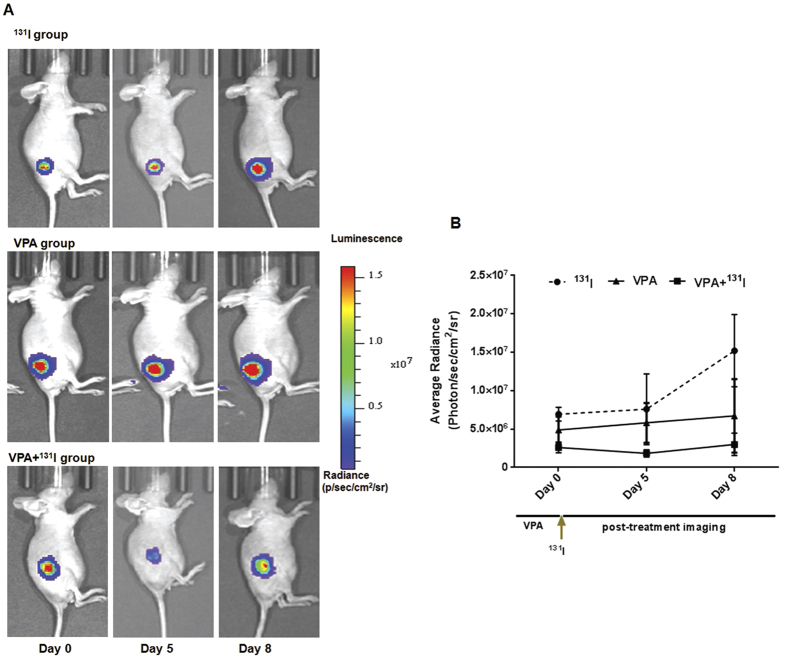
Non-invasive bioluminescence monitoring of Zr-75-1 tumor xenograft bearing mice during the treatment phase. (**A**) Tumor xenografts were established in nude mice using engineered Zr-75-1-CMV-Fluc2.Tdt reporter cells. Mice in ^131^I group received single intraperitoneal injection of 1mCi of ^131^I on day 0, and mice in VPA group received 5 doses of 250 mg/kg VPA treatment alone while the experimental group undergone pre-treatment with 250 mg/kg VPA for 5 days before ^131^I treatment started. (**B**) Graph illustrates mean bioluminescence signal quantities from sets of mice (n = 3) at day 0, day 5 and day 8 when the mice were scanned by injecting D-luciferin.

Further, ^131^I therapeutic potential *in vivo* was also tested, where ^131^I radioablative treatment efficacy was measured by noninvasive bioluminescence imaging. Here we used Zr-75-1 cells over-expressing luciferase reporter (Fluc2.tdTomato fusion) so that change in tumor growth can be monitored non-invasively. The results reveal that in mice treated with ^131^I (1 mCi/mouse) or 5 doses of VPA (250 mg/kg) alone, tumors continue to grow, whereas mice pre-treated with VPA and ^131^I showed 30% lesser bioluminescence signal 5 days after treatment (Fig. 6A,B). As lowered luciferase signal is a direct measure of tumor burden, these first hand results seem encouraging indicating the scope of using HDACi treatment to enhance ^131^I therapeutic efficacy in breast cancer.

## Discussion

**Figure 7:**
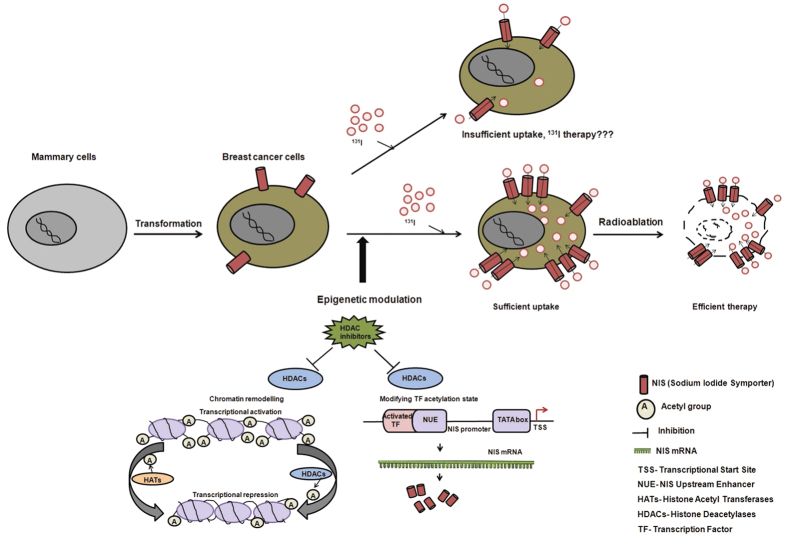
Schematic illustration of potential therapeutic applications of HDACi mediated human NIS modulation in breast cancer. Upregulation of NIS expression during malignant transformation of breast tissue implicated therapeutic application of radioiodine in breast cancer management. To achieve enhanced therapeutic effect, stimulation of functional NIS expression by epigenetic modifiers such as HDACi is a promising strategy.

Over the last fifteen years, several studies have reported NIS expression in BC raising the possibility of NIS gene-targeted radionuclide imaging and therapy. Although moderate NIS expression is encountered in majority of BC cases, sufficient radioiodide accumulation has been found in a very small fraction of cases. Therefore, selective induction of NIS protein expression in breast tissue has appeared to be an important strategy towards achieving this goal. In the present study, we have tested a wide panel of HDAC inhibitors for transcriptional modulation of human NIS in breast cancer cells. As revealed in the schematic diagram (Fig. 7), in addition to histone acetylation activity, distinct HDACi may display different biological responses by acting through various transcriptional factors that in turn can alter human NIS expression. So in the panel of drugs, we have included TSA, TBA, and SAHA which are derivatives of hydroxamic acids, NaB and VPA which are short chain fatty acids and CI994 which is a new benzamide compound. Importantly, most of these drugs are pan-DAC inhibitors and also in phase 2 or 3 clinical trial (Table 1), except for TBA which was included in our study as a HDAC6 specific inhibitor. To our knowledge, this is the first report evaluating such wide scale HDACi class of molecules for altering NIS expression in breast cancer as well as several other non-thyroid and thyroid cancer cell types. As the results reflect, four out of six HDACi especially the candidate drugs like NaB, VPA, CI994, and SAHA have shown promising induction in NIS transcript and protein expression.

Focusing on breast cancer, one key aspect of our study is the differential modulation observed in endogenous NIS expression of hormone receptor positive cells than receptor negative cells. Until now enhancement of NIS expression, function and various signaling pathways involved are studied primarily in ER-positive cells^23^,^24^,^25^. Triple negative tumors (ER, PR, HER2 –ve), on the other hand, have worst prognosis and are in high demand of targeted and systemic therapy options. A significant number of TNBC tumor samples showing intense NIS staining^8^, can now be modulated further by HDACi, and thus eventually may improve the scope of applying NIS-based targeted radioiodide therapy for this subgroup of patients. NIS mRNA induction is found much more pronounced in estrogen receptor (ER) positive cells than the ER-negative cells. Recent clinical finding reported by us shows that in breast cancer NIS expression has strong association with ER expression among all the BC subtypes^8^. As NIS promoter has several estrogen response elements (revealed by bioinformatics analysis), estrogen is expected to have a direct role in driving NIS transcription. HDACi are also known to act on nuclear receptors including ERα^26^. But surprisingly, in our TF array, ER activation and binding to NIS promoter was not pronounced (i.e. above threshold cutoff margin). Thus, future studies will have to confirm the regulatory mechanism by which HDACi treatment cause enhanced effect in ER positive cells.

Another important aspect of this study is the information elucidated from the TF activation and promoter binding array of 96 major factors. These results show for the first time that candidate TFs which potentially play regulatory role on NIS expression in BC cells. NaB and VPA treatment in MCF-7 cell results in mostly differential activation of TFs except for Stat4 being the only common candidate found in place. Further, *in silico* analysis (using Genomatix and Transfac Software) as well as promoter binding array results provide confirmation on the presence of TF binding sites on NIS promoter (as listed in Table 2) supporting their potential direct role in modulating cellular NIS expression. In thyroid, it is well established that TSH stimulation activates cAMP and CREB resulting in activation of NIS expression^27^. But the role of CREB in regulation of NIS in BC is still unknown. PPAR ligands in combination with other agents were known to induce NIS expression in thyroid tissue^28^. There is no literature available regarding regulation of NIS expression by various TFs like AP1, Stat4 etc. as turned out to be important factors in our results. These observations put forward important mechanistic insights on human NIS promoter induction indicating role of specific TFs in breast tissue which can be explored further in future.

Further, augmented NIS protein levels after NaB, VPA or CI994 treatment are also found to be sufficient for ^131^I radioablation in culture. Increased *in vivo* uptake of NIS-mediated ^99m^Tc-pertechnetate is also confirmed using MDA-MB-231 tumor xenograft and gamma camera imaging. Also, together with the data on ^131^I therapy in cultured cell, mouse studies using noninvasive bioluminescence imaging has clearly indicated HDACi treatment improved therapeutic benefit *in vivo*. HDACis are expected to show anti-proliferative effect to some extent at the delivered dose in mouse. Therefore, for further significant improvement of NIS-mediated therapeutic efficacy, dose optimization of VPA and ^131^I can be tried in future. Effective thyroid blocking may also improve therapeutic efficacy as more ^131^I become available in the body. For successful implementation of endogenous NIS mediated gene therapy approach, the major bottlenecks are insufficient radioiodide accumulation in the tumor bed as well as short tracer retention time, putting a question mark on adequate therapeutic value of this approach. In cultured plate, our results suggest that although HDACi treatment significantly improves NIS-mediated iodide uptake, iodide efflux remains impervious. At this point, there is no proven strategy that shows organification of iodine in BC cells can happen^1^, and thus whether or not this process can be tapped to enhance efficacy is a matter of research in future. For effective radioiodide therapy, the membrane localization of NIS protein is a critical factor along with its total expression on the cell. From our study, we have found that treatment of HDACi significantly increases NIS expression but it doesn’t alter its localization (Fig. 3D). Even though NIS protein expression has been observed in 70% of the breast cancer patients, its clinical utility has been undermined due the intracellular localization of the protein in majority cases. Therefore for successful therapeutic intervention, finding out potential agents/signalling pathways targeting NIS protein to the cell the membrane should also be explored in future.

In conclusion, to the best of our knowledge, this is the first study that provides important mechanistic insights on HDACi mediated NIS over-expression in breast cancer cell. This study also demonstrates *in vivo* functional improvement as a step forward towards taking this gene therapy strategy to a success. In breast cancer cases, where NIS protein over-expresses upon cancer onset, use of a brief HDACi pre-treatment may turn NIS mediated radioiodide therapy efficacious and thus seems to be a promising strategy for future clinical application. Apart from breast cancer, in thyroid and other non-thyroidal cancer types, where NIS transgene mediated therapy has often been used, may also be impacted further by this transcriptional modulation strategy.

## Materials and Methods

### Chemicals and Cell lines

HDACi drugs such as TSA [T8852], NaB [B5887], VPA [4543], SAHA [SML0061], TBA [SML0044] and all trans retinoic acid [R2625] were purchased from Sigma, USA. CI994 (1742-10, 50) was purchased from Biovision, CA, USA. Stock solutions (1M) of TSA, SAHA, CI994 and TBA were prepared in DMSO and of NaB and VPA were prepared in sterile MiliQ and stored at −20 °C. Receptor positive MCF-7 and Zr-75-1 BC cells (ATCC) were maintained in RPMI-1640 media (Gibco, Invitrogen, USA). Receptor negative MDA-MB-231 cells (ATCC) were maintained in Leibovitz’s L15 media (Sigma, USA) whereas MDA-MB-468 cells (a gift from Dr. M. Vaidya, ACTREC, India) were maintained in DMEM media (Gibco, Invitrogen, USA) supplemented with 10 mM HEPES. All the media contained 10% fetal bovine serum (FBS) (Gibco, Invitrogen, USA), 1% antibiotic-antimycotic solution (Gibco, Invitrogen, USA). Two thyroid cancer cell lines, NPA and ARO (gifted by Mr. A. Chakraborty, BARC, India), were maintained in IMDM (Gibco, Invitrogen, USA) containing 10% FBS and 0.075% gentamycin solution. All the cells were maintained at 37 °C in a humidified incubator (Thermo Scientific, Rockford, IL, USA) with 5% CO_2_ except for MDA-MB-231.

### Plasmid Construction

pGL3-NIS-luc+ vector containing 1.34 kb human NIS promoter was a kind gift from Dr. Kenneth Ain, University of Kentucky, USA. Human NIS promoter was PCR amplified using primers (5′-GGCACGCGTATGTGCCACCACG and 3′-GGCGCTAGCGGAGGTCGCCTTG) with 5′-MluI and 3′-NheI enzyme sites and cloned into pcDNA3.1(+) mammalian expression vector containing Fluc2.TurboFP bi-fusion reporter. Positive clones were confirmed by PCR amplification, restriction digestion and sequencing. CMV-TurboFP.Rluc8.6 expression vector was developed as control plasmid using NheI and BamHI restriction sites.

### Stable cell lines

The pNIS-Fluc2.TurboFP plasmid DNA was transfected into MCF-7, MDA-MB-231 cells using lipofectamine 2000 (Life Technologies, USA). Clonal selection was achieved using G418 sulphate (500 μg/ml for MCF-7 and MDA-MB-231) (Sigma, USA). Positive clones were confirmed by luciferase reporter activity as well as TurboFP fluorescence expression.

### MTT cell cytotoxicity assay

To evaluate cytotoxicity of various HDACi, MCF-7 and MDA-MB-231 (5 × 10^3^) cells were seeded in 96 well plates (Corning, USA). Cells were exposed to different concentrations of HDACi for 48 hours. Cell viability was assessed using the MTT (3-[4,5-dimethylthiazol-2-yl]c-2,5-diphenyltetrazolium bromide) (Sigma, USA) as cell proliferation reagent.

### RNA extraction and quantitative real-time PCR

After the cells were treated with HDACi for 48 hours, RNA was extracted using RNeasy kit (QIAGEN, USA). cDNAs were synthesized using the first strand cDNA synthesis kit (Invitrogen, USA). Quantitative real-time PCR was performed using Taqman probe mix on the 7900HT PCR cycler (Applied Biosystems, USA). The Taqman probes for human NIS and GAPDH with assay ids Hs00166567_m1 and Hs02758991_g1 respectively were used (Applied Biosystems, USA). Triplicate samples were run for each sample. The comparative ΔΔCt method was used to calculate relative gene expression.

### Immunoblotting and immunofluorescence procedures

Cells were treated with various HDACi for 48 hours and were lysed using cell lysis buffer (Cell Signalling Technology, USA) containing standard protease inhibitors (Santa Cruz Biotechnology, USA). Equal amount of protein from control and treated cells were resolved in 10% SDS-PAGE gel and transferred onto nitrocellulose membrane (Millipore, USA) by semi-dry method. After blocking with 5% BSA (Bovine Serum Albumin), membranes were probed with respective monoclonal antibodies, such as anti-acetyl-histone H3 antibody (06-598, Upstate, USA) and anti-Fluc antibody (G4751, Promega Corporation, USA), or anti-human NIS antibody (FP5A, Abcam, USA), and anti-α-tubulin antibody (T9026, Sigma, USA) followed by washing and secondary HRP-conjugated antibodies. The blot was developed by chemiluminescence (Invitrogen, USA). To determine even transfer and equal loading, membranes were stripped and re-probed with an α-tubulin monoclonal as per standard.

For immunofluorescence study, cells were fixed with 4% paraformaldehyde, washed with PBS and blocked with 2% BSA followed by overnight incubation with primary antibody for human NIS (FP5A, Abcam, USA) and γH2Ax (Pierce Biotechnology, USA) at 4 °C. The cells were then washed with PBS, incubated with anti-mouse (NIS)/ anti rabbit (γH2Ax) Dylight 633 conjugated secondary antibody (Thermo Scientific, Rockford, IL, USA) (1h), washed and counterstained for nucleus using DAPI (Sigma, USA). Fluorescence micrographs were captured using Carl Zeiss LSM 510 Meta confocal microscope.

### Luciferase reporter assay

Selected MCF-7 and MDA-MB-231 clonal cell population were seeded and treated with different HDACi for 48 hours. Lysates were collected using passive lysis buffer (Promega Corporation, USA) and luciferase activity was measured using luminescence plate reader (BMG Labtech, Germany).

### Transcription factor (TF) activation profiling and NIS promoter binding array

We used a 96 well plate TF activation array (# FA1002, Signosis, USA) as per the manufacturer’s guideline. MCF-7 cells were treated with NaB/ VPA for 48 hours and nuclear proteins from both control and treated cells were isolated using standard procedure and used as probe on the array plate. Transcription factor IID (TFIID) was used for normalization of readings. TFs were selected by considering the fold-change method (≤1.5 or ≥1.5) of treated over untreated cell samples. To characterize binding of various activated TFs to NIS promoter, promoter binding TF profiling array was performed (FA2002, Signosis, USA). Nuclear extracts were prepared from NaB/ VPA treated MCF-7 cells, incubated with oligo-binding mix along with NIS-promoter DNA fragment. Comparing luminescence in presence or absence of competitor human NIS promoter, binding of various TFs were predicted.

### Iodide uptake and efflux measurement assays

Iodide uptake and efflux study was performed as described previously^29^,^30^ with minor modifications. After 48 hours of treatment with indicated HDACis, cells were incubated with 10 μM NaI in uptake buffer [Hank’s Balanced Salt Solution (HBSS) supplemented with 10 mM HEPES (pH 7.3)]. To determine NIS-specific iodide uptake, cells were incubated with 30 μM KClO4 in uptake buffer for one hour prior to addition of 10 μM NaI. After 30 minutes incubation with NaI at 37 °C, cells were washed with ice-cold uptake buffer. Then 10.5 mM ammonium cerium (IV) sulphate solution and 24 mM sodium arsenite (III) solution were added. The plate was incubated at room temperature (RT) in dark for 30 minutes and the absorbance at 420nm was recorded. Using logarithmic conversion and standard equation of iodide standards, amount of nanomoles (nmoles) of iodide uptake was calculated from absorbance read-outs.

In iodide efflux measurement study, control or drug treated cells were incubated in uptake buffer with 10 μM NaI at 37 °C for 30 minutes. Cells were washed with ice cold uptake buffer to stop the reaction and fresh uptake buffer without NaI was added. The medium was replaced after every 10 minutes and 10.5 mM ammonium cerium (IV) sulphate solution and 24 mM sodium arsenite (III) solution were added. Following incubation at RT in dark for 30 minutes absorbance at 420nm was recorded.

### *In vitro* clonogenic assay

Control and NaB/ VPA/ CI994 treated MCF-7/ Zr-75-1/ MDA-MB-231 cells were grown in 25 cm^2^ flasks and incubated with 50 μCi/ml of ^131^I in case of MCF-7 or MDA-MB-231 and 100 μCi/ml in case of Zr-75-1 in HBSS supplemented with 10 μM NaI and 10 mM HEPES (pH7.3). The *in vitro* clonogenic assay was performed as described by Mandell *et al*.^29^.

### *In vivo* preclinical imaging

The experimental protocol was approved by Institutional Animal Ethics Committee at KNU, Korea and performed in accordance with the guidelines for the Care and Use of the Laboratory Animals. The MDA-MB-231 cells (5 × 10^6^) were implanted along with matrigel (4:1) in 6-week-old BALB/c nude mice subcutaneously. When tumor reached ~300 mm^3^ volume, mice were divided into three groups (n = 3): the control (intraperitoneal injection of 100 μL PBS), VPA (250 mg/kg) and VPA (500 mg/kg). Pre- and post-treatment gamma camera imaging using a pinhole collimator (Infinia II, GE Healthcare, USA) was conducted at 20 minutes after ^99m^Tc-pertechnetate (10.0 MBq) injection. The mice were maintained under isoflurane (Forane®, ChoongWae Co., Seoul, Korea) anesthesia during the entire process. The same procedure was also used for the NaB treatment (500 mg/kg and 1000 mg/kg body weight).

### ^125^I autoradiography of tumor xenograft

The experimental protocol was approved by Institutional Animal Ethics Committee at KNU, Korea and performed in accordance with the guidelines for the Care and Use of the Laboratory Animals. Autoradiography of MDA-MB-231 tumor xenograft was performed for NIS mediated radioiodine uptake. Mice with grown tumor were segregated in three groups, i.e. control, VPA treated and NaB treated (n = 4 in each group). After VPA (250 mg/kg) and NaB (500 mg/kg) treatment for 5 days and 24 hours of last drug treatment, all mice were injected with 50–60 μCi ^125^ I via the tail vein. Four hours post injection, mice were sacrificed and tumors were excised and prepared for standard autoradiography procedure. Then frozen sections of 20 μm thickness were cut using cryostat and mounted on glass slides. The glass slides were exposed in the imaging cassette for 24 h; subsequently, the exposed plate was scanned with a film imaging analyzer (Fuji film, FLA-3000) to confirm the uptake. ROI measurements were done from the captured digital images and compared between treatment groups with that of the vehicle treated control.

### *In vivo* optical imaging and immunohistochemistry procedures

The experimental protocol was approved by Institutional Animal Ethics Committee (IAEC) at ACTREC and performed in accordance with the guidelines for the Care and Use of the Laboratory Animals using ACTREC animal house and Molecular Imaging facilities. Female BALB/c nude mice (n = 9) were used for growing subcutaneous tumor using Zr-75-1 cells labeled with Fluc2.Tdt fusion gene. Mice were divided into three groups: the ^131^I group (intraperitoneal injection of 1mCi Na-^131^I on day 0) , VPA group (5 doses of 250 mg/kg of VPA alone) and the experimental group (5 doses of 250 mg/Kg of VPA followed by 1mCi Na-^131^I on day 0). Serial Bioluminescence imaging was performed using IVIS-Spectrum (Caliper Life Sciences) after intraperitoneal injecting 30 mg/ml of D-luciferin (Biosynth International) and viewed in real time on a computer screen using a color scale expressed as total flux (photons per second per square centimeter per steradian [photons/sec/cm^2^/sr]). Mice were anesthetized with isofluorane and placed in the imaging chamber with continuous 2% isofluorane administration via nasal cone. Data were analyzed using Living Image version 4.4 software.

For immunohistochemistry (IHC), tumors from drug treated (5 daily doses of 250 mg/kg) and control group were harvested and fixed using standard procedures^8^. For digital scoring of IHC slides, we used the IHCprofiler plugin for ImageJ (software) developed by our group^31^.

### Statistical Analysis

All data are expressed as mean ± SE and are representative of at least two separate experiments. Statistical significance was analyzed by Student t-test using GraphPad Prism 6 (GraphPad Software, La Jolla, CA). P values of ≤0.05 were considered statistically significant.

## Additional Information

**How to cite this article**: Kelkar, M. G. *et al*. Enhancement of human sodium iodide symporter gene therapy for breast cancer by HDAC inhibitor mediated transcriptional modulation. *Sci. Rep.* **6**, 19341; doi: 10.1038/srep19341 (2016).

## Supplementary Material

Supplementary Information

## Figures and Tables

**Table 1:** Various HDAC inhibitors used in this study to check their effect on NIS expression.

Group	Compound	HDAC Targets	Potency (in cells)	Type of cancer	Clinical Trial	Reference
Hydroxymate	Trichostatin A (TSA)	Class I, II	Nano Molar	—	—	—
	Suberoylanilide hydroxamic acid (SAHA)	Class I, II	Micro Molar	CTCL	FDA approval for CTCL	32
	Tubastatin A (TBA)	HDAC6	—	—	—	—
Aliphatic acid	Valproic acid (VPA)	Class I & IIa	Milli Molar	Prostate cancer	Phase 2	33
	Sodium butyrate (NaB)	Class I & IIa	Milli Molar	Lung cancer	Phase 2	34
Benzamide	CI994	NA	Micro Molar	Pancreatic cancer	Phase 2	35

NA- Not Available.

FDA- Food and Drug Administration.

CTCL- Cutaneous T Cell Lymphoma.

**Table 2:** List of transcription factors that are activated and binding to NIS promoter after NaB/VPA treatment.

Transcription factor (TF)	Response to HDACi tested	Putative Binding Sequence on NIS promoter as per Transfac/Genomatix analysis	No. of sites	Involved in NIS induction in breast or thyroid cancer	Reference
CREB	NaB	gaagCGTGAcccccc	5	Thyroid	27
AP1	VPA	cggTGACCCggga	1	ND	
GATA	VPA	ggcacTTATCa	6	ND	
NF-1	VPA	ctggcacaggGCCAAct	2	ND	
PPAR	VPA	accagaacctccAGAGgtcaaag	2	Thyroid	28
Hox4C	VPA	tggtctcCGCTtattcggg ^#^	6	ND	
Stat4/Stat6	NaB, VPA	tcgaaTTCTGgga ^#^	1	ND	
Sox9	NaB	aggagccAATGaatgaatgaatgaa ^#^	2	ND	
E2F-1	NaB	gctgctcccgtaagccccaaGGCGacctc	1	ND	

^#^indicates presence of TF binding site belonging to family of TF i.e. Hox, Stat and Sox family of TFs respectively.

ND- role in NIS regulation is not determined.
