# Supplementary material for: Enhancement of human sodium iodide symporter gene therapy for breast cancer by HDAC inhibitor mediated transcriptional modulation
Source: Sci Rep. 2016 Jan 18;6:19341. doi: 10.1038/srep19341 (PMC4726020; doi:10.1038/srep19341)
Supplement: Supplementary Information [file srep19341-s1.docx]

**Enhancement of human sodium iodide symporter gene therapy for breast cancer by HDAC inhibitor mediated transcriptional modulation**

**Madhura G. Kelkar1, Kalimuthu Senthilkumar 2, Smita Jadhav1, Sudeep Gupta3, Beyong-Cheol Ahn2 and Abhijit De1**

**1** Molecular Functional Imaging Lab, ACTREC, Tata Memorial Centre, Kharghar, Navi Mumbai, India.

**2** Department of Nuclear Medicine, Kyungpook National University, Republic of Korea.

**3** Department of Medical Oncology, Tata Memorial Hospital, Parel, Mumbai, India.

***Correspondence:**

Dr. Abhijit De

Molecular Functional Imaging Lab

Advanced Centre for Treatment, Research and Education in Cancer (ACTREC)

Tata Memorial Centre

Sector 22, Kharghar

Navi Mumbai – 410210

Maharashtra, India

Phone: +91-22-2740 5038

FAX: +91-22-2740 5085

Email: ade@actrec.gov.in

**Authors Emails:** MK <madhuragk@yahoo.com>; KS <senthilbhus@gmail.com>; SJ < smitajadhav20@gmail.com >; SG <sudeepgupta04@yahoo.com>; BCA <abc2000@knu.ac.kr>

**Supplementary Material:**

**
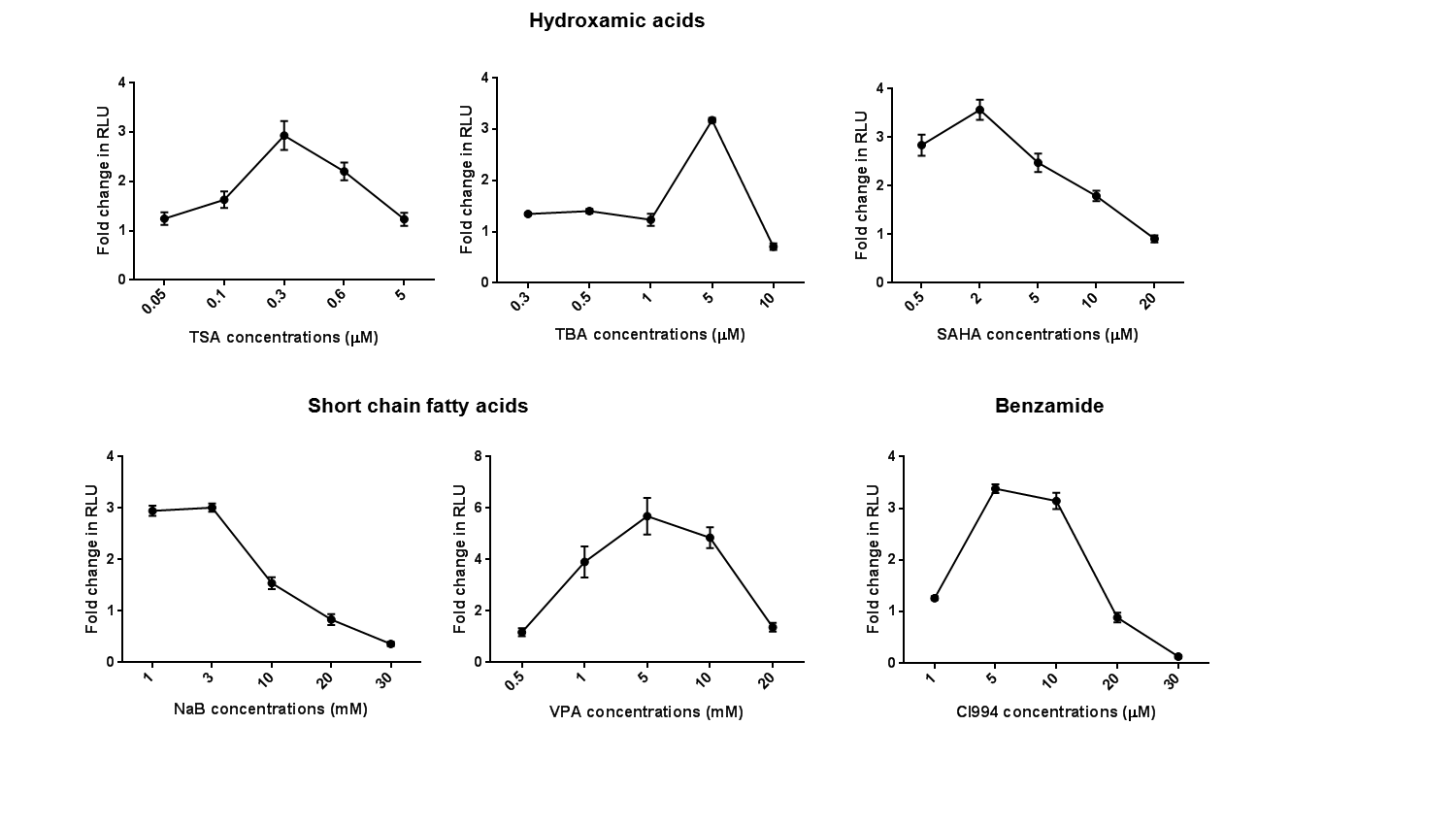
**

**Figure S1:** Dose dependent effect of various HDACi on NIS promoter in MCF-7 cells stably expressing NIS-promoter driven luciferase reporter. Treatment was given for 48 hours and fold change in luciferase activity as compared to untreated cells were computed. The concentration of HDACi showing highest fold increase in NIS promoter activity was chosen as optimal drug concentration for promoter activation and used for all other experiments.


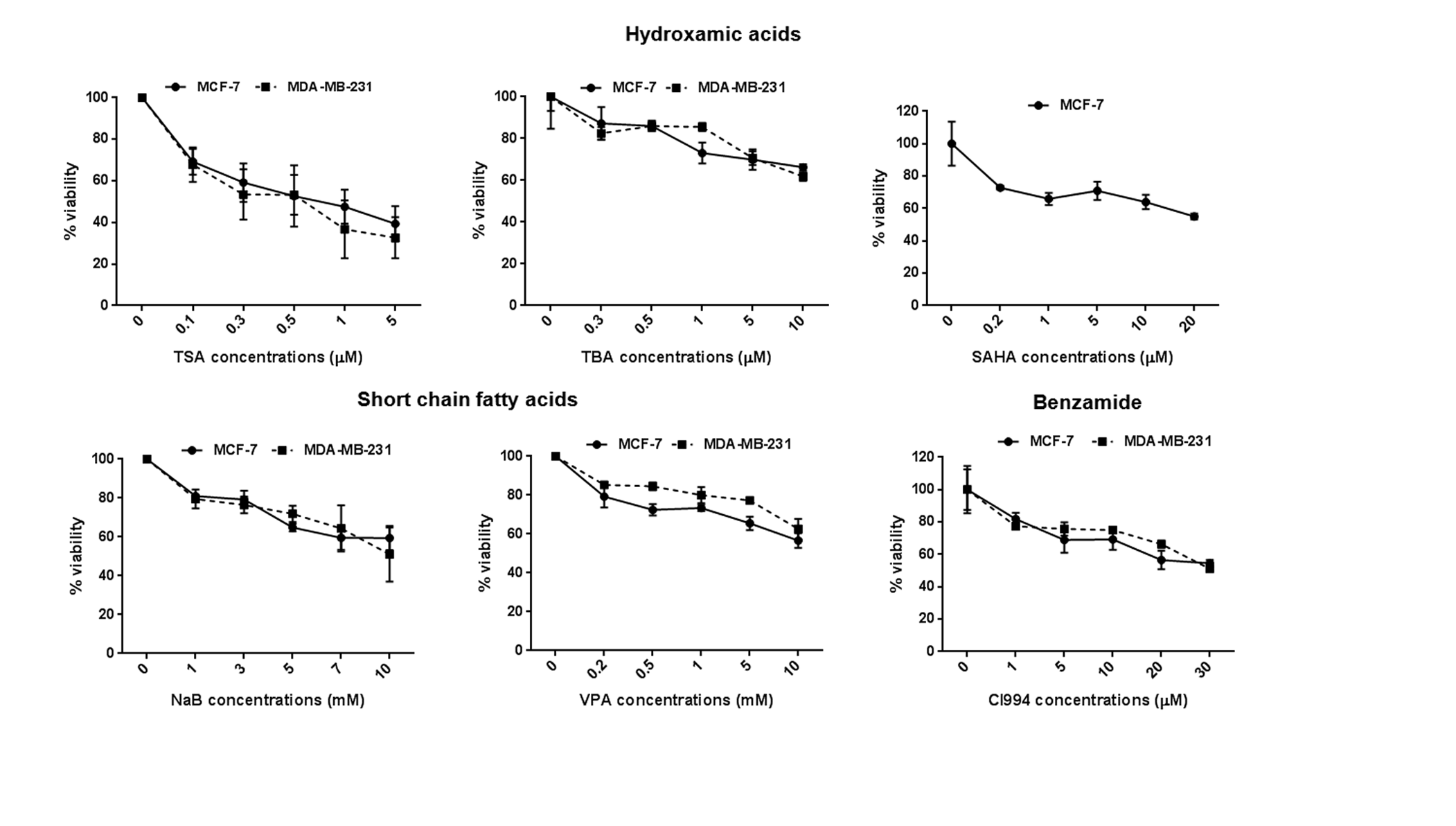


**Figure S2:** Study of cytotoxicity of HDACi treatment in MCF-7 and MDA-MB-231 breast cancer cells. Cells were incubated with different doses of HDACi for 48 hours and cell viability was measured by the MTT assay. Data represented as percentage viability to untreated controls.


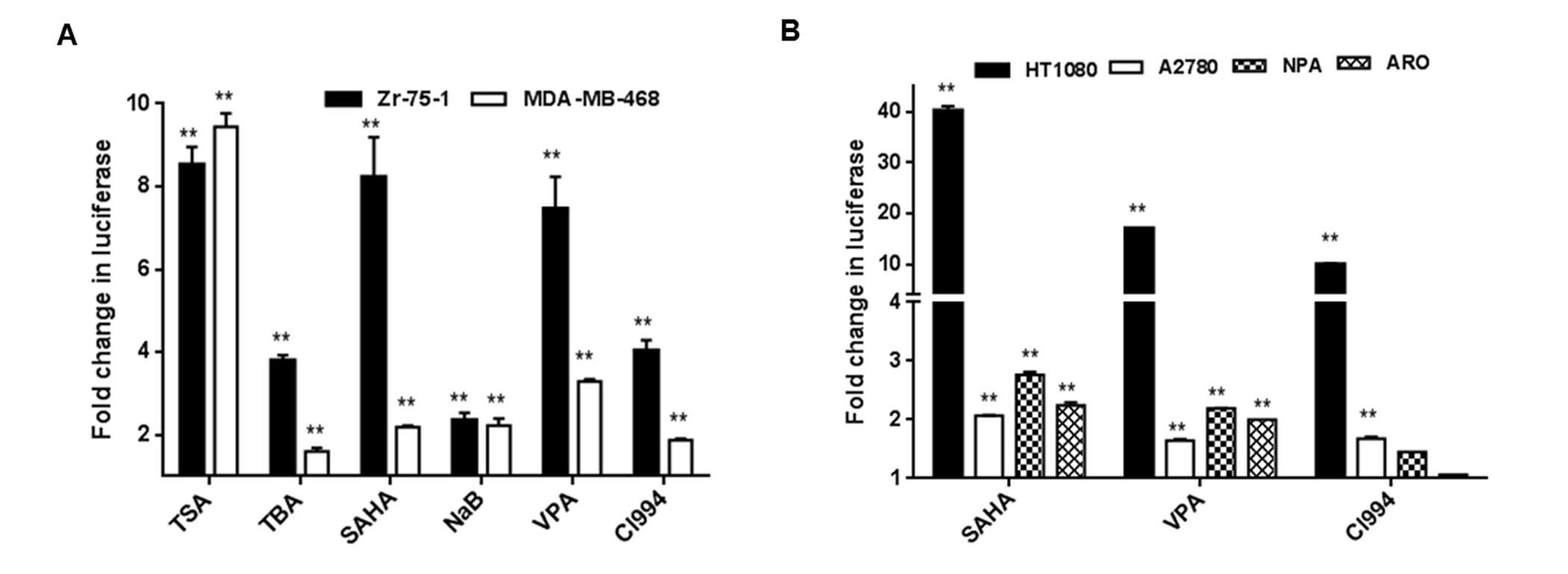


**Figure S3:** Study of HDACi treatment on promoter specificity and luciferase expression in multiple cancer cell lines. All cell lines were transiently transfected with pNIS FLuc2.TurboFP plasmid and normalized by pCMV-TurboFP.Rluc8.6 plasmid. **(A)**. Chart represents fold change in photon values over untreated control cell in two additional breast cancer cell lines. **(B)**. One representative drug from each chemical class of HDACi was tested in non-breast cancer cell lines.

**
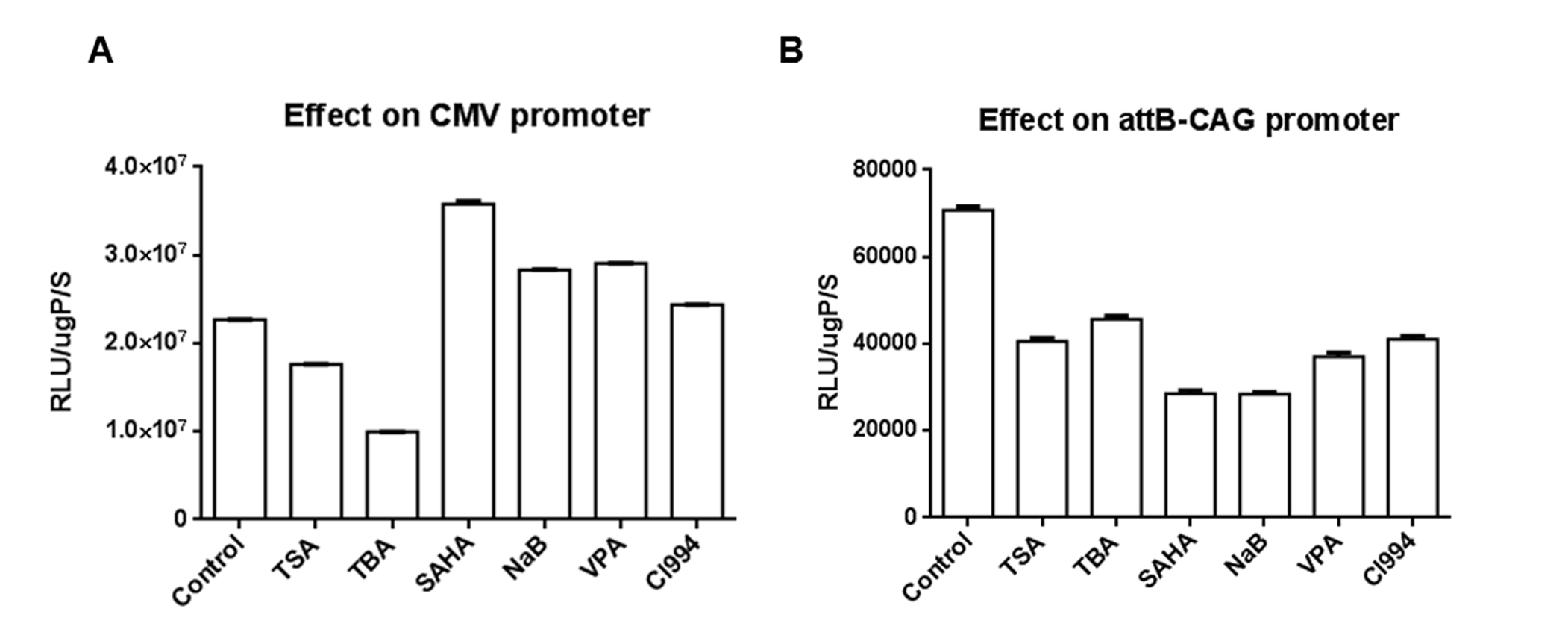
**

**Figure S4:** Study of promoter specific effect of HDACi treatment. Effect of six HDACi was tested on two other promoters i.e. **(A)** cytomegalovirus (CMV) and **(B)** chicken β-actin (CAG). MCF-7 cells stably over-expressing mammalian expression plasmids with luciferase reporter cloned under the respective promoter used for measuring drug effect on the promoters.


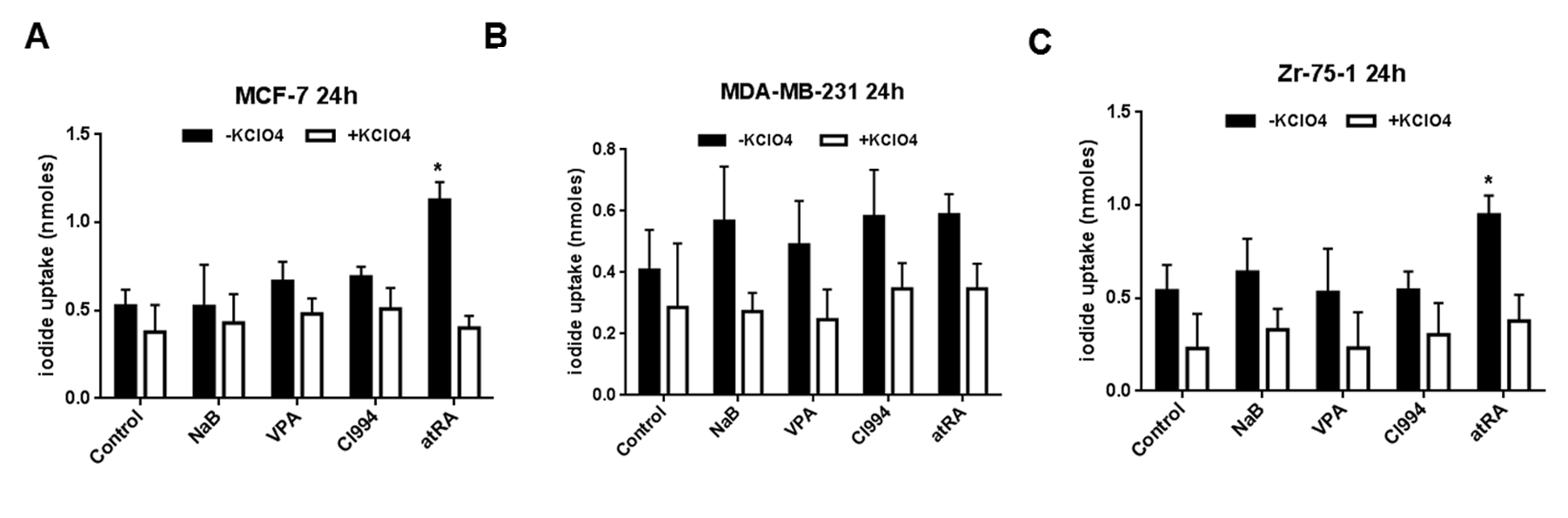


**Figure S5:** Comparison of effect of atRA and HDACi on NIS-mediated iodide uptake in breast cancer cells. MCF-7 (**A**), MDA-MB-231 (**B**) and Zr-75-1 (**C**) cells were treated with NaB, VPA, CI994 and atRA for 24 hours respectively. Effect of HDACi was compared with the effect of atRA in all the three cell lines. KClO4 was used as a competitive inhibitor of NIS function. Error bars indicate standard error of mean. (* indicates high significance p<0.05)


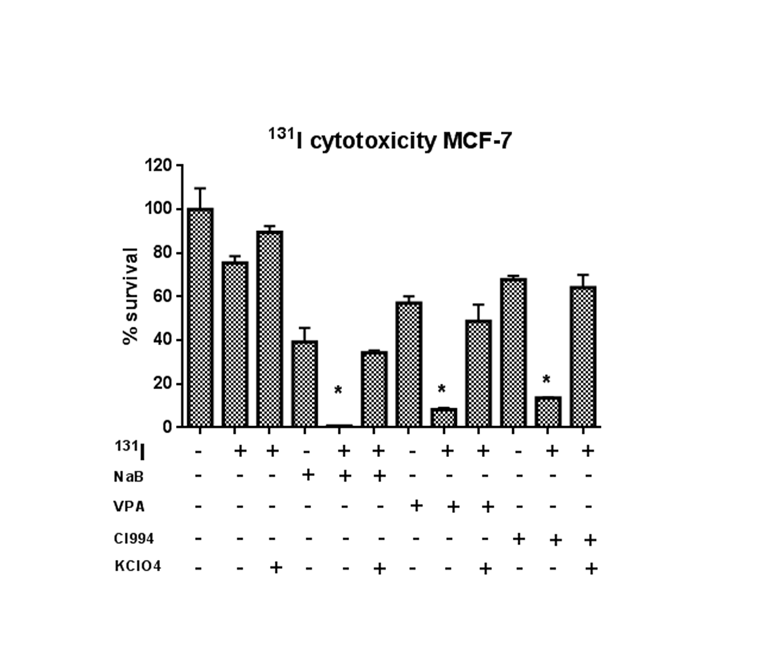


**Figure S6:** ^131^I mediated cytotoxicity assay in MCF-7 cells in presence of KClO4 blocking agent. Therapeutic efficacy of ^131^I radioablation was verified in MCF-7 cells after NaB, VPA and CI994 pre-treatment in the presence or absence of blocking agent KClO4. (* indicates high significance p<0.05).

**
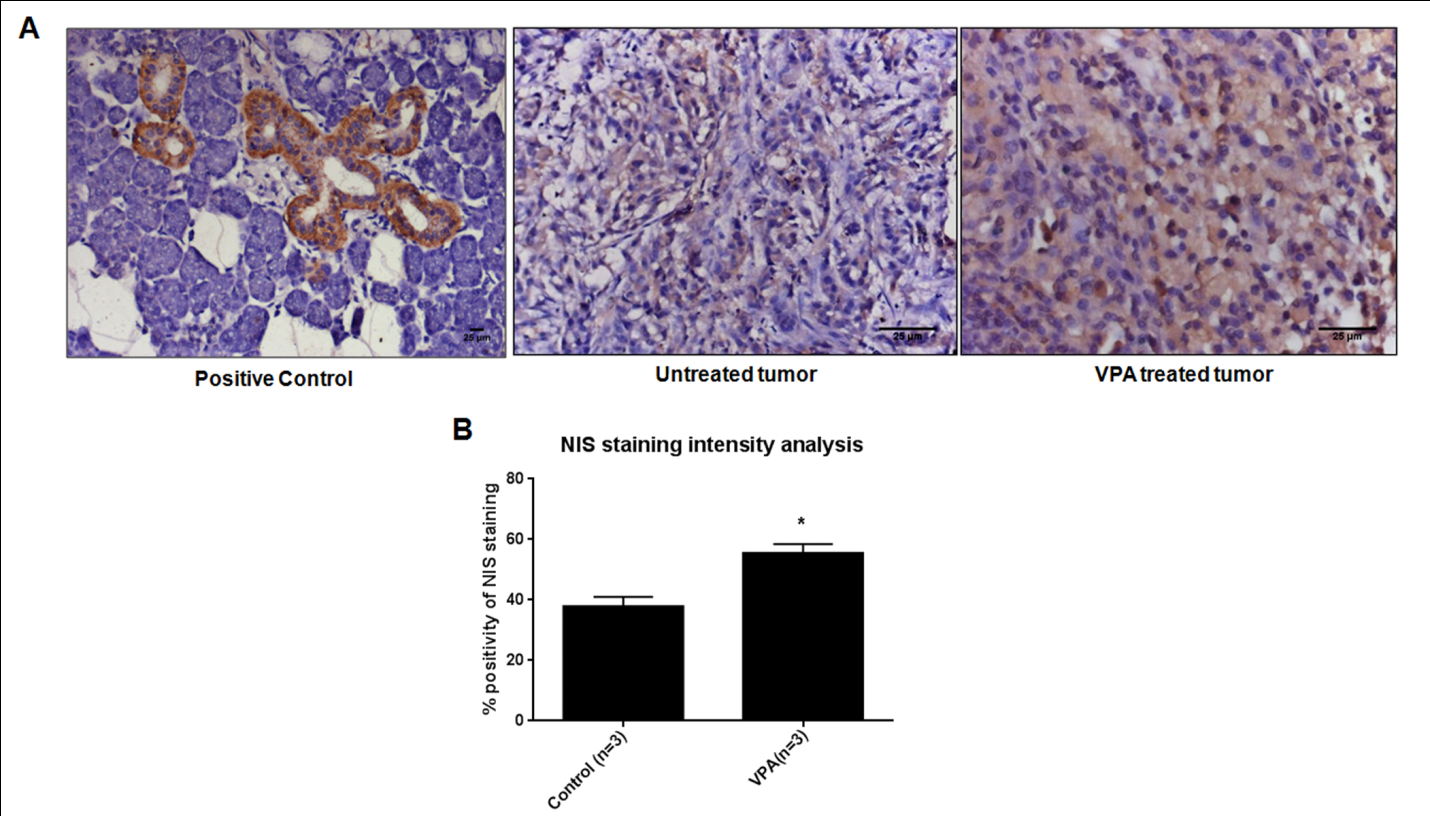
**

**Figure S7:** Analysis of NIS protein expression after VPA treatment Zr-75-1 tumor xenografts. **(A)**. Representative photomicrograph of NIS antibody stained Zr-75-1 tumor xenografts with or without VPA treatment. As positive control, a normal human salivary gland tissue section was used. All images were captured using 20X magnification. **(B)**. Chart represents NIS staining intensity comparison of treated and untreated tumor samples with an average of 12 digital sampling collected from multiple xenografts. * indicates statistical significance with p<0.05.


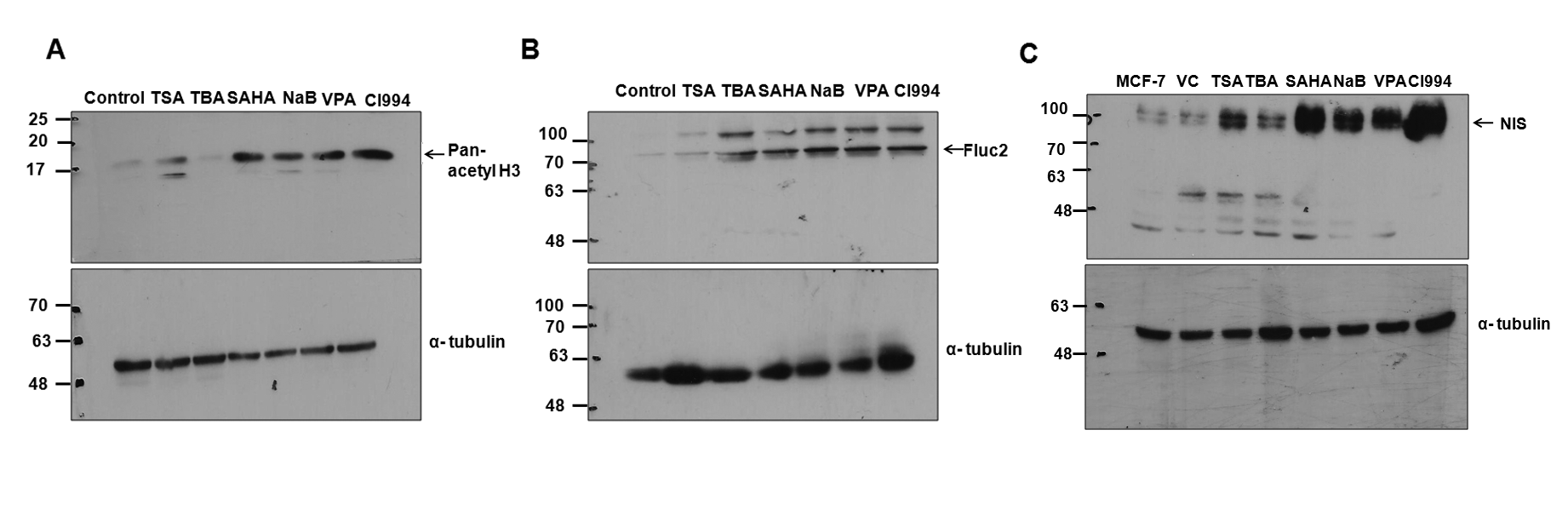


**Figure S8:** Full length western blots for acetyl-histone H3 (**A**), Fluc2 (**B**) and human NIS (**C**) in MCF-7 cells. α-tubulin was used as a loading control. The bands indicated with arrows were presented in the main figure.
